# Drug Transporter Genetic Variants Are Not Associated with TDF-Related Renal Dysfunction in Patients with HIV-1 Infection: A Pharmacogenetic Study

**DOI:** 10.1371/journal.pone.0141931

**Published:** 2015-11-04

**Authors:** Takeshi Nishijima, Tsunefusa Hayashida, Takuma Kurosawa, Noriko Tanaka, Shinichi Oka, Hiroyuki Gatanaga

**Affiliations:** 1 AIDS Clinical Center, National Center for Global Health and Medicine, Tokyo, Japan; 2 Center for AIDS Research, Kumamoto University, Kumamoto, Japan; 3 Department of Mathematical Science for Information Sciences, Graduate School of Science, Tokyo University of Science, Tokyo, Japan; 4 Biostatistics Section, Department of Clinical Research and Informatics, Clinical Science Center, National Center for Global Health and Medicine, Tokyo, Japan; Commissariat a l'Energie Atomique(cea), FRANCE

## Abstract

**Objective:**

To investigate whether single nucleotide polymorphisms (SNP) of drug transporter proteins for TDF is a risk factor for TDF-related renal function decrement.

**Methods:**

This study investigated the association between 3 SNPs (*ABCC2*–24, 1249, and *ABCB1* 2677), which are shown to be associated with TDF-induced tubulopathy, and clinically important renal outcomes (>10ml/min/1.73m^2^ decrement in eGFR relative to baseline, >25% decrement in eGFR, and eGFR <60ml/min/1.73m^2^) in 703 HIV-1-infected Japanese patients who initiated TDF-containing antiretroviral therapy (ART). Genotyping was performed by allelic discrimination using TaqMan 5’-nuclease assays.

**Results:**

95% of the study patients were males and 66% were treatment-naïve, with median CD4 count of 249/μl, median baseline eGFR of 96ml/min/1.73m^2^ (IQR 84.6–109.2), and median exposure to TDF of 3.66 years (IQR 1.93–5.59). The frequencies of genotypes at -24, 1249 of *ABCC2*, and 2677 of *ABCB1* were neither different between patients with decrement in eGFR of >10ml/min/1.73m^2^ and those without such decrement (*ABCC2*: -24, p = 0.53, 1249, p = 0.68; *ABCB1*: 2677, p = 0.74), nor between those without and with the other two renal outcomes (>25% decrement: *ABCC2*: -24, p = 0.83, 1249, p = 0.97, *ABCB1*: 2677, p = 0.40; eGFR <60ml/min/1.73m^2^: *ABCC2*: -24, p = 0.51, 1249, p = 0.81, *ABCB1*: 2677, p = 0.94). Logistic regression analysis showed that the risk genotype of the three SNPs were not associated with any of the three renal outcomes, respectively. Logistic regression model that applied either dominant, recessive, or additive model yielded the same results.

**Conclusions:**

SNPs of the drug transporters for TDF are not associated with clinically important renal outcomes in patients who initiated TDF-containing ART.

## Introduction

Tenofovir disoproxil fumarate (TDF), a prodrug of tenofovir, is one of the most widely used nucleotide reverse transcriptase inhibitors (NRTI) for the treatment of HIV-1 infection in both resource-rich and resource-limited settings [1,2], and also for the treatment of hepatitis B infection [3]. Furthermore, the use of TDF, either as a fixed dose with emtricitabine (FTC) or alone, has been recently recommended by the WHO and American CDC guidelines, as pre-exposure prophylaxis for prevention of transmission of HIV-1 in high-risk uninfected adults [4,5].

Tenofovir is predominantly excreted by the kidney through the combination of glomerular filtration and active tubular secretion [6]. TDF is known to cause renal proximal tubular dysfunction, such as Fanconi’s syndrome [7], and also reduces the estimated glomerular filtration rate (eGFR) more than other NRTIs [8,9]. Although the exact mechanism of tenofovir-induced nephrotoxicity is not fully understood, mitochondria toxicity in proximal renal tubular cells has been suspected as the main cause of TDF-related renal function decrement [10].

Because the severity of tenofovir nephrotoxicity varies widely among individuals, the role of host genetics has drawn a particular attention. Many single nucleotide polymorphisms (SNPs) of the genes encoding transporter proteins in renal tubular cells, such as organic anion transporter (OAT) 1 and 3, multidrug resistance protein (MRP) 2, 4, and 7, and P-glycoprotein, have been investigated to elucidate their roles in tenofovir-induced tubulopathy [11–15]. As a result, genotype C/C at -24 (rs717620) and genotype A/A at 1249 (rs2273697) on the *ABCC2* gene, which encode MRP2, consistently shown the association with tenofovir-induced tubulopathy [11,13,16]. However, whether individuals with such SNPs are more susceptible to TDF-related renal function decrement than those without such genetic variants remains to be elucidated. This issue is important because HIV-1 infection requires life-long antiretroviral therapy (ART) and renal dysfunction and chronic kidney diseases are important comorbidities that can influence mortality [17,18].

Based on the above background, the present study was designed to elucidate the association between polymorphisms in genes encoding drug transporters in renal tubular cells and tenofovir-related renal function decrement among HIV-1-infected patients who initiated TDF-containing ART.

## Methods

### Ethics Statement

This study was approved by the Human Genetics Research Ethics Committee of the National Center for Global Health and Medicine, Tokyo, Japan. Each patient included in this study provided a written informed consent for genetic testing and publication of clinical data for research purposes. The study was also conducted according to the principles expressed in the Declaration of Helsinki.

### Study Design and subjects

We performed a single-center cohort study to investigate the association between TDF-related renal function decrement and SNPs in genes encoding renal tubular transporters in Japanese HIV-1-infected patients who initiated TDF-containing ART. The inclusion criteria for the study patients were: 1) Japanese patients with HIV infection who initiated TDF-containing ART at our clinic between January 2002 and December 2013, 2) patients who continued TDF for ≥90 days, and 3) patients who provided a written informed consent for the study. Patients with eGFR <60 ml/min/1.73m^2^ at initiation of TDF were excluded. The written informed consent was obtained from the candidate patients between June 2014 and October 2014.

### Measurements

The eGFR was calculated using the equation developed by the Japanese Society of Nephrology (JSN): eGFR = 194 × [serum creatinine]^-1.094^ × [age]^-0.287^ × [0.739 if female] [19]. This equation is used because this is more suitable for patients with small body stature, such as Japanese, than The Chronic Kidney Disease Epidemiology Collaboration (CKD-EPI) equation [20,21]. The 2013 practice guidelines for patients with CKD published by JSN also recommend the use of this equation for the Japanese, rather than the CKD-EPI equation [21]. The baseline eGFR was estimated for each patient from age, sex, and serum creatinine measurements made closest to and preceding the initiation of ART by no more than 90 days. Patients visited our clinic at least every three months for monitoring CD4 cell count, HIV-1 viral load, and eGFR, since the prescription period under the Japanese health care system is limited to three months [22]. Thus, for calculation of follow-up eGFR values, we collected serum creatinine values measured closest to every 90 day within a range of 45 days from initiation of ART. Follow up eGFR data were collected from the baseline measurement until discontinuation of TDF or at the end of the follow-up period (August 2014).

The potential risk factors for renal dysfunction were determined according to previous studies and collected together with the basic demographics from the medical records [23–26]. They included age, sex, body weight, body mass index (BMI) = {body weight (kg) / [(height (m)]^2^}, history of AIDS, route of HIV-1 transmission, HIV-1 treatment status (either treatment-naïve or experienced), combination of ART, baseline laboratory data (CD4 cell count, HIV viral load, and serum creatinine), and presence or absence of other medical conditions [diabetes mellitus defined by using anti-diabetic agents or fasting plasma glucose >126 mg/dl or plasma glucose >200 mg/dl on two different days, hypertension defined by current treatment with antihypertensive agents or two successive measurements of systolic blood pressure >140 mmHg or diastolic blood pressure >90 mmHg at the clinic, dyslipidemia defined by current treatment with lipid-lowering agents, co-infection with hepatitis B defined by positive hepatitis B surface antigen, co-infection with hepatitis C defined by positive HCV viral load, and current smoking] [22], concurrent use of ritonavir-boosted PIs (PI/r), concurrent use of nephrotoxic drugs, such as ganciclovir and sulfamethoxazole/trimethoprim. At our clinic, body weight and blood pressure were measured on every visit whereas other variables were measured in the first visit and at least once annually [22]. We used the data on or closest to and preceding the day of starting ART by no more than 180 days.

### Genetic polymorphisms

The selected SNPs were *1)* -24C→T (in the promoter; rs717620) and *2)* 1249G→A (Val417Ile; rs2273697) of *ABCC2* gene, because they are the only SNPs that have consistently shown close association with tenofovir-induced tubulopathy in previous studies [11,13,16]. In addition, 2677T→A/G (A:Ser893Thr, G:Ser893Ala; rs2032582) of *ABCB1* gene, which encodes P-glycoprotein, was also selected, because this triallelic SNP is functionally significant and appears to influence the absorption of TDF at the intestine and affect exposure of tenofovir [6,27,28].

### Pharmacogenetic analyses

Genomic DNA was extracted from peripheral blood leukocytes using the QIAamp DNA MiniKit and the protocol provided by the manufacturer (Qiagen, Valencia, CA). All genotyping was performed by allelic discrimination using TaqMan 5’-nuclease assays with standard protocols (TaqMan SNP Genotyping Assays; Applied Biosystems, Foster City, CA). The primer and probe sequences are available on request.

### Statistical analysis

Three renal endpoints were applied in this study; we focused primarily on 1) decrement in eGFR of >10 ml/min/1.73 m^2^ relative to the baseline, because this endpoint is considered appropriate for patients with well maintained renal function [22,29], such as the study population. We also set two commonly used renal endpoints; 2) >25% decrement in eGFR relative to the baseline [30,31], and 3) eGFR <60 ml/min/1.73 m^2^ [32].

The baseline characteristics were compared between patients with decrement in eGFR of >10 ml/min/1.73 m^2^ and those without such decrement by the Student’s t-test or the Wilcoxon signed-rank test for continuous variables and by either the χ^2^ test or Fisher’s exact test for categorical variables. The χ^2^ test was used to test for deviation of allele frequency from the Hardy-Weinberg equilibrium. Statistical comparisons for genotype frequencies between the two groups were tested by the Fisher’s exact test or the χ^2^ test where appropriate. The logistic regression model was used to estimate the association of risk genotype/allele of each SNP with the occurrence of these renal endpoints. We applied the following four genetic models for the analysis: genotype model (a model that postulates no mode of inheritance), dominant model, recessive model, and additive model. Each genetic effect in logistic regression models was estimated with the adjustment for the variables which were determined a priori; they included baseline eGFR, age, baseline body weight, nephrotoxic drug use, PI/r use, CD4 count, hypertension, and dyslipidemia, which are established risk factors for TDF nephrotoxicity [9,23,24,26]. Sex and diabetes mellitus were not added to the models due to their low frequency. The above statistical analyses were repeated using eGFR calculated by the CKD-EPI equation adjusted with the Japanese coefficient [33].

Statistical significance was defined at two-sided *p* values <0.05. We used the odds ratio (OR) with 95% confidence intervals (95% CI) as a measure of the effect of risk allele/genotype on each renal endpoint. All statistical analyses were performed with SAS software, version 9.3 (SAS Institute, Cary, NC).

## Results

**Fig 1:**
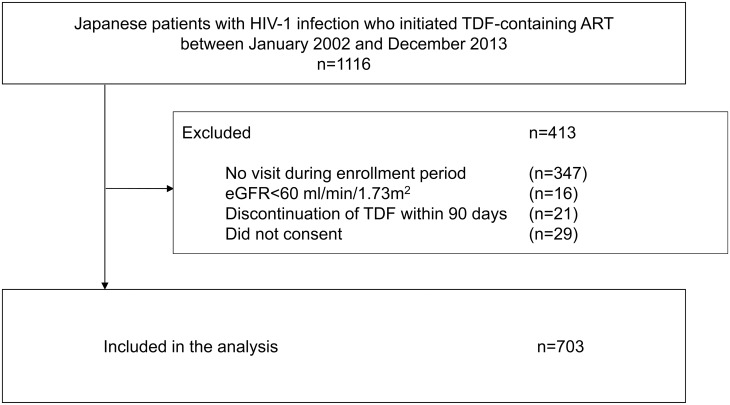
Flow diagram of the patient enrolment process.

A total of 703 patients who satisfied the inclusion and exclusion criteria and provided a written informed consent during the inclusion period were enrolled in the study (Fig 1). The study patients were mostly homosexual male with a median age of 38 (IQR 33–46) (Table 1). The median CD4 count at baseline was 249 /μL (IQR 127–385), and 66% of the study patients were treatment-naïve for HIV-1 infection. With regard to ART, 75% of the patients started TDF with PI/r. The median baseline eGFR was 96 ml/min/1.73 m^2^ (IQR 84.6–109.2) [by CKD-EPI equation: 94.2 ml/min/1.73 m^2^ (88.3–100.3)]. The median duration of TDF use was 3.66 years (IQR 1.93–5.59).

**Table 1:** Baseline characteristics of the study patients.

	Study patients (n = 703)	>10 ml/min/1.73 m^2^ decrement (n = 624)	No decrement in eGFR (n = 79)	P value
Sex (male), n (%)	669 (95)	592 (95)	77 (97)	0.41
Age^†^	38 (33–46)	38 (33–46)	39 (34–47)	0.18
Weight (kg)^†^	63.3 (57.3–70)	63 (57–70)	66 (60–73)	0.077
BMI (kg/m^2^)^†^	22 (20.2–24.1)	21.9 (20–24.1)	22.8 (20.9–24.2)	0.26
eGFR: JCKD-EPI (ml/min/1.73m^2^)^†^	94.2 (88.3–100.3)	95.4 (89–101.1)	88.4 (76.8–92.4)	<0.0001
eGFR: JeGFR (ml/min/1.73m^2^)^†^	96 (84.6–109.2)	98.2 (86.7–112.2)	82.1 (68.3–91.6)	<0.0001
Serum creatinine (mg/dl)^†^	0.74 (0.65–0.82)	0.72 (0.65–0.80)	0.84 (0.79–0.96)	<0.0001
CD4 count (/μl)^†^	249 (127–385)	244.5 (110–377.5)	304 (188–436)	0.0058
HIV RNA viral load (log_10_/ml)^†^	4.51 (2.66–5.11)	4.5 (2.6–5.1)	4.4 (2.8–4.9)	0.52
Treatment naive, n (%)	467 (66)	416 (67)	51 (64)	0.71
Ritonavir-boosted protease inhibitors, n (%)	529 (75)	470 (75)	59 (75)	0.89
Protease inhibitors (unboosted), n (%)	22 (3)			
NNRTIs, n (%)	71 (10)			
INSTIs, n (%)	89 (13)			
Hypertension, n (%)	110 (16)	93 (14)	17 (22)	0.14
Dyslipidemia, n (%)	311 (44)	274 (44)	37 (47)	0.63
Diabetes mellitus, n (%)	19 (3)	18 (3)	1 (1)	0.71
Concurrent use of nephrotoxic drugs, n (%)	110 (16)	99 (16)	11 (14)	0.74
Hepatitis B, n (%)	79 (11)	77 (12)	2 (3)	0.0070
Hepatitis C, n (%)	35 (5)	34 (5)	1 (1)	0.16
History of AIDS, n (%)	207 (29)	188 (30)	19 (24)	0.30
Homosexual contact, n (%)	565 (80)			
Injection drug user	7 (1)			
Current smoker, n (%)	307 (44)	275 (44)	32 (41)	0.63
TDF duration (years)^†^	3.66 (1.93–5.59)	3.89 (2.14–5.67)	1.52 (0.96–3.15)	<0.0001

^†^median (interquartile range)

Nine patients were taking both PI/r and NNRTI, 1 patient with NNRIT and INSTI. 1 patient was treated with 2 NRTIs and 1 with 3 NRTIs. Other patients were treated with 2 NRTIs together with either PI, NNRTI, or INSTI.

Differences between the two groups were compared by the Student’s t-test for continuous variables and by Fisher’s exact test for categorical variables, except for CD4 count, HIV RNA viral load, and TDF duration, which were compared by the Wilcoxon signed-rank test.

BMI: body mass index, TDF: tenofovir disoproxil fumarate, eGFR: estimated glomerular filtration rate, NNRTI: non- nucleoside reverse transcriptase inhibitor, INSTI: integrase strand transfer inhibitor.

Of the 703 study patients, >10 ml/min/1.73 m^2^ decrement in eGFR relative to the baseline occurred in 624 (89%), >25% decrement in 119 (17%), and eGFR <60 ml/min/1.73 m^2^ in 126 (18%). Patients with >10 ml/min/1.73 m^2^ decrement in eGFR had higher baseline eGFR (p<0.0001), lower CD4 count (p = 0.0058), had more frequent HBV co-infection (p = 0.0070), and had longer exposure to TDF (p<0.0001), compared to those without decrement in eGFR (Table 1).

Table 2 summarizes the distribution of genotypes at -24 and 1249 of *ABCC2* gene and at 2677 of *ABCB1* gene, for patients with each renal endpoint and those free of decrement in eGFR. All polymorphisms were in Hardy-Weinberg equilibrium. The frequencies of genotypes at -24, 1249 of *ABCC2* gene and at 2677 of *ABCB1* gene were not different between patients with >10 ml/min/1.73 m^2^ decrement in eGFR and those without decrement in eGFR (-24 of *ABCC*2, p = 0.53, 1249 of *ABCC2*, p = 0.68, 2677 of *ABCB1*, p = 0.74), between patients with >25% decrement in eGFR and those without (-24 of *ABCC*2, p = 0.83, 1249 of *ABCC2*, p = 0.97, 2677 of *ABCB1*, p = 0.40), and between patients with decrement in eGFR to <60 ml/min/1.73 m^2^ and those without (-24 of *ABCC*2, p = 0.51, 1249 of *ABCC2*, p = 0.81, 2677 of *ABCB1*, p = 0.94).

**Table 2:** Genotype frequencies at three SNPs of *ABCC2* and *ABCB1* in patients with and without three renal outcomes.

		>10 ml/min/1.73 m^2^ decrement in eGFR from baseline			>25% decrement in eGFR from baseline			eGFR <60 ml/min/1.73 m^2^		
	Amino acid	>10 ml/min/1.73 m^2^ decrement (n = 624)	No decrement (n = 79)	P value*	>25% decrement (n = 119)	No decrement (n = 584)	P value*	<60 ml/min/1.73 m^2^ (n = 126)	No decrement (n = 577)	P value*
*ABCC2* (MRP2)										
-24 C→T, rs717620										
C/C		382 (61)	51 (65)		76 (64)	357 (61)		83 (66)	350 (61)	
C/T		215 (35)	27 (34)	0.53	38 (32)	204 (35)	0.83	38 (30)	204 (35)	0.51
T/T		27 (4)	1 (1)		5 (4)	23 (4)		5 (4)	23 (4)	
1249 G→A, rs2273697	Val417Ile									
G/G		483 (78)	61 (77)		93 (78)	451 (77)		100 (79)	444 (77)	
A/G		132 (21)	16 (20)	0.68	24 (20)	124 (21)	0.97	24 (19)	124 (21)	0.81
A/A		9 (1)	2 (3)		2 (2)	9 (2)		2 (2)	9 (2)	
*ABCB1* (P-glycoprotein)										
2677T→A/G, rs2032582	A:Ser893Thr G:Ser893Ala									
T/T		112 (18)	13 (16)		19 (16)	106 (18)		21 (17)	104 (18)	
T/A		77 (12)	13 (16)		22 (18)	68 (11)		18 (14)	72 (12)	
G/G		122 (20)	13 (16)	0.74	20 (17)	115 (20)	0.40	21 (17)	114 (20)	0.94
G/T		195 (31)	29 (37)		39 (33)	185 (32)		41 (32)	183 (32)	
G/A		96 (15)	9 (12)		17 (14)	88 (15)		20 (16)	85 (15)	
A/A		22 (4)	2 (3)		2 (2)	22 (4)		5 (4)	19 (3)	

*By use of Fisher’s exact test for 2×3 table (2×6 table for rs2032582).

The results of additional analyses using eGFR calculated by the CKD-EPI equation are shown in Table 3. Similarly, the frequencies of genotypes at -24, 1249 of *ABCC2* gene and at 2677 of *ABCB1* gene were not different between patients with each renal endpoint and those who reached no such endpoint (>10 ml/min/1.73 m^2^ decrement in eGFR: -24 of *ABCC*2, p = 0.59, 1249 of *ABCC2*, p = 0.20, 2677 of *ABCB1*, p = 0.95) (>25% decrement in eGFR: -24 of *ABCC*2, p = 0.62, 1249 of *ABCC2*, p = 0.86, 2677 of *ABCB1*, p = 0.22) (eGFR <60 ml/min/1.73 m^2^: -24 of *ABCC*2, p = 0.91, 1249 of *ABCC2*, p = 1.00, 2677 of *ABCB1*, p = 0.76).

**Table 3:** Genotype frequencies of three SNPs of *ABCC2* and *ABCB1* in patients with and without three renal outcomes calculated by the CKD-EPI equation.

		>10 ml/min/1.73 m2 decrement in eGFR from baseline			>25% decrement in eGFR from baseline			eGFR <60 ml/min/1.73 m2		
	Amino acid	>10 ml/min/1.73 m^2^ decrement (n = 624)	No decrement (n = 79)	P value*	>25% decrement (n = 119)	No decrement (n = 584)	P value*	<60 ml/min/1.73 m^2^ (n = 126)	No decrement (n = 577)	P value*
*ABCC2* (MRP2)										
-24 C→T, rs717620										
C/C		302 (62)	131 (61)		79 (64)	354 (61)		38 (66)	395 (61)	
C/T		166 (34)	76 (36)	0.59	39 (31)	203 (35)	0.62	18 (31)	224 (35)	0.91
T/T		22 (4)	6 (3)		6 (5)	22 (4)		2 (3)	26 (4)	
1249 G→A, rs2273697	Val417Ile									
G/G		386 (79)	158 (74)		98 (79)	446 (77)		45 (78)	499 (77)	
A/G		95 (19)	53 (25)	0.20	24 (19)	124 (21)	0.86	12 (21)	136 (21)	1.00
A/A		9 (2)	2 (1)		2 (2)	9 (2)		1 (1)	10 (2)	
*ABCB1* (P-glycoprotein)										
2677T→A/G, rs2032582	A:Ser893Thr G:Ser893Ala									
T/T		83 (17)	42 (20)		19 (15)	106 (18)		9 (15)	116 (18)	
T/A		62 (13)	28 (13)		24 (19)	66 (11)		8 (14)	82 (13)	
G/G		95 (19)	40 (19)	0.95	21 (17)	114 (20)	0.22	12 (21)	123 (19)	0.76
G/T		157 (32)	67 (31)		41 (33)	183 (32)		15 (26)	209 (32)	
G/A		75 (15)	30 (14)		17 (14)	88 (15)		12 (21)	93 (14)	
A/A		18 (4)	6 (3)		2 (2)	22 (4)		2 (3)	22 (4)	

*By use of Fisher’s exact test for 2×3 table (2×6 table for rs2032582).

The logistic regression model which evaluated genotypic effect of the *ABCC2* gene showed that the risk genotype (i.e., genotype CC at -24) was not associated with any of the three renal outcomes (Table 4) (>10 ml/min/1.73 m^2^ decrement in eGFR: genotype C/C versus T/T, adjusted OR 0.5, 95%CI 0.06–3.91, p = 0.62; genotype C/T versus T/T, adjusted OR 0.4, 95%CI 0.05–3.33, p = 0.34) (>25% decrement in eGFR: genotype C/C versus T/T, adjusted OR 1.2, 95%CI 0.42–3.20, p = 0.62; genotype C/T versus T/T, adjusted OR 1.0, 95%CI 0.35–2.83, p = 0.81) (eGFR <60 ml/min/1.73 m^2^: genotype C/C versus T/T, adjusted OR 0.6, 95%CI 0.20–2.08, p = 0.61; genotype C/T versus T/T, adjusted OR 0.6, 95%CI 0.17–1.93, p = 0.36). Similarly, the risk genotype (genotype A/A) at 1249 of *ABCC2* or a genotype at 2677 of *ABCB1* was not associated with either of the three renal outcomes (S1 and S2 Tables). Furthermore, logistic regression analysis, which applied the dominant model, recessive model, and additive model, showed no association between each allele/genotype at the SNPs and any of the three renal endpoints (S3 Table). Logistic regression analysis using eGFR calculated by the CKD-EPI equation yielded the same results. Post-hoc analysis with the logistic models after further adjustment for duration of TDF therapy also yielded the same results.

**Table 4:** Effects of SNP at -24 of *ABCC2* on three renal outcomes in patients who initiated TDF-containing antiretroviral therapy: Multivariate logistic regression with genotype model.

	>10 ml/min/1.73 m^2^ decrement in eGFR			>25% decrement in eGFR			eGFR<60 ml/min/1.73 m^2^		
	OR	95%CI	P value	OR	95%CI	P value	OR	95%CI	P value
Genotype C/C versus T/T	0.5	0.06–3.91	0.62	1.2	0.42–3.20	0.62	0.6	0.20–2.08	0.61
Genotype C/T versus T/T	0.4	0.05–3.33	0.34	1.0	0.35–2.83	0.81	0.6	0.17–1.93	0.36

Odds ratios for each genotype were adjusted for baseline eGFR, age, CD4 count, body weight, nephrotoxic drug use, hypertension, dyslipidemia, and use of PI/r. OR: odds ratio, CI: confidence interval, eGFR: estimated glomerular filtration rate, PI/r: ritonavir-boosted protease inhibitor.

## Discussion

This pharmacogenetics study investigated the association between drug transporter genetic variants and TDF-related renal function decrement in Japanese HIV-1-infected patients who initiated TDF-containing ART. The results showed that none of the three examined SNPs was associated with any of the three selected renal outcomes: >10 ml/min/1.73 m^2^ decrement in eGFR relative to the baseline, >25% decrement in eGFR, and eGFR <60 ml/min/1.73 m^2^. The results were reproduced using the dominant, recessive, and additive models, in addition to the genotype model for the estimation of association between genetic variants and renal outcomes.

Two main aspects of our study are important. First, this study showed that genetic factors do not need to be taken into account as predisposing factors for TDF–related renal dysfunction, using three clinically important renal outcomes (>10 ml/min/1.73 m^2^ decrement in eGFR relative to the baseline [22,29], >25% decrement [30,31], and eGFR <60 ml/min/1.73 m^2^ [32], which are known to be associated with morbidity and mortality in HIV-1-infected patients [17,18]. In this regard, one study from Thailand reported the association between -24 C/C genotype of *ABCC2* gene and lower eGFR in treatment-naïve patients who initiated TDF-containing non-NRTI based regimen [34]. However, the relatively small sample size of 117 patients and, more importantly, the cross-sectional analysis used for the assessment of the association between eGFR and genotype at 48 and 96 weeks undermine the reliability of their findings, because in using such design, the value of eGFR at 48 or 96 weeks is inevitably affected by the baseline eGFR. Furthermore, another recent Thai study of 238 patients showed that SNPs of drug transporters, including -24 and 1249 of *ABCC2* gene, were not associated with a change in creatinine clearance from the baseline to 1 and 3 years of TDF exposure [35]. Nevertheless, our sample size is the largest (n = 703) among the studies investigating the effect of genetic variants of drug transporters on TDF-related renal dysfunction, and by using clinically relevant renal outcomes, our study showed that SNPs were not associated with TDF nephrotoxicity.

Second, to our knowledge, this is the first study to apply not only genotype model (a model that postulates no mode of inheritance), but also dominant, recessive, and additive models, to investigate the association between genetic variants and TDF nephrotoxicity [16,34,35]. It is noteworthy that none of the four genetic models showed any association between genetic variants of transporter proteins and TDF-associated renal dysfunction, especially considering that it is unknown which genetic model is appropriate for the evaluation of the effect of SNPs on TDF nephrotoxicity. Another strength of this study is that the results were reproduced with eGFR calculated by the CKD-EPI equation, in addition to the results based on eGFR calculated by the JSN equation.

It is noteworthy that although the association between the SNPs of *ABCC2* investigated in this study and TDF-induced tubulopathy has been well established [11,13,16], the exact mechanism by which these SNPs pose a risk for TDF tubulopathy remains unknown [13,16]. In this regard, MRP2 encoded by *ABCC2* is not likely to take part in the transportation of TDF at the luminal membrane of kidney tubular cells [16,36]. At this point, because the results of this study showed that genetic variants of the drug transporters for TDF are not associated with clinically important renal outcomes, we think that these SNPs do not count as a risk factor for TDF-related renal dysfunction, at least in the clinical setting, and efforts should rather be focused on the management of traditional risk factors for renal dysfunction, such as diabetes mellitus and hypertension [37], as well as the management of PI/r, antiretroviral agents that are reported to increase TDF exposure [38] and thus are a risk factor for TDF-related renal dysfunction [23]. It is also notable that among PI/r, ritonavir-boosted atazanavir and lopinavir/ritonavir are reported to be associated with CKD [39].

Several limitations need to be acknowledged. First, the patients who discontinued TDF within 90 days from the initiation of this therapy were excluded from the study. It is difficult to completely exclude the possibility that inclusion of such patients would have resulted in misleading results, because the subjects would have included some who experienced substantial decrement in renal function due to causes other than TDF, such as death shortly after initiation of ART or immune reconstitution inflammatory syndrome of opportunistic infections, considering that two-third of the study patients were treatment-naive. Second, although we selected the target SNPs that have been identified to associate with TDF-induced tubulopathy reported in previous studies, there might be other unknown transporter proteins for tenofovir excretion or transportation that contribute to susceptibility to tenofovir nephrotoxicity. Third, this study did not measure TDF plasma concentration, which could correlate with TDF-induced renal dysfunction [40]. Fourth, our cohort was characterized by the high prevalence of PI/r use, which can affect plasma concentration of TDF [41], and it is difficult to completely exclude the impact of concurrent PI/r in this study.

In conclusion, the present study demonstrated that genetic variants of the drug transporters for TDF do not associate with clinically important renal outcomes in patients who started TDF-containing ART. Such SNPs are not considered to be a risk factor for clinically relevant TDF-related renal dysfunction.

## Supporting Information

S1 Table Effects of SNP at 1249 of *ABCC2* on three renal outcomes in patients who initiated TDF-containing antiretroviral therapy: Multivariate logistic regression with genotype model. (DOCX)

S2 Table Effects of SNP at 2677 of ABCB1 on three renal outcomes in patients who initiated TDF-containing antiretroviral therapy: Multivariate logistic regression with genotype model. (DOCX)

S3 Table Effects of SNPs on three renal endpoints using dominant, recessive, and additive genetic models for multivariate logistic analysis. (DOCX)
