# Supplementary material for: Drug Transporter Genetic Variants Are Not Associated with TDF-Related Renal Dysfunction in Patients with HIV-1 Infection: A Pharmacogenetic Study
Source: PLoS One. 2015 Nov 4;10(11):e0141931. doi: 10.1371/journal.pone.0141931 (PMC4633171; doi:10.1371/journal.pone.0141931)
Supplement: S1 Table — (DOCX) [file pone.0141931.s001.docx]

S1 Table 1. Effects of SNP at 1249 of *ABCC2* on three renal outcomes in patients who initiated TDF-containing antiretroviral therapy: Multivariate logistic regression with genotype model.

|  | >10 ml/min/1.73 m^2^ decrement in eGFR | | |  | >25% decrement in eGFR | | |  | eGFR <60 ml/min/1.73 m^2^ | | |
| --- | --- | --- | --- | --- | --- | --- | --- | --- | --- | --- | --- |
|  | OR | 95%CI | P value |  | OR | 95%CI | P value |  | OR | 95%CI | P value |
| Genotype A/A versus G/G | 0.3 | 0.05-1.77 | 0.22 |  | 1.2 | 0.24-5.57 | 0.82 |  | 2.3 | 0.38-14.2 | 0.34 |
| Genotype A/G versus G/G | 0.8 | 0.42-1.54 | 0.46 |  | 0.9 | 0.57-1.55 | 0.77 |  | 0.9 | 0.51-1.66 | 0.34 |

Odds ratios for each genotype were adjusted for baseline eGFR, age, CD4 count, body weight, nephrotoxic drug use, hypertension, dyslipidemia, and use of PI/r. OR: odds ratio, CI: confidence interval, eGFR: estimated glomerular filtration rate, PI/r: ritonavir-boosted protease inhibitor.
