# Supplementary material for: Drug Transporter Genetic Variants Are Not Associated with TDF-Related Renal Dysfunction in Patients with HIV-1 Infection: A Pharmacogenetic Study
Source: PLoS One. 2015 Nov 4;10(11):e0141931. doi: 10.1371/journal.pone.0141931 (PMC4633171; doi:10.1371/journal.pone.0141931)
Supplement: S2 Table — (DOCX) [file pone.0141931.s002.docx]

S2 Table 2. Effects of SNP at 2677 of *ABCB1* on three renal outcomes in patients who initiated TDF-containing antiretroviral therapy: Multivariate logistic regression with genotype model.

|  | OR | 95%CI | P value |  | OR | 95%CI | P value |  | OR | 95%CI | P value |
| --- | --- | --- | --- | --- | --- | --- | --- | --- | --- | --- | --- |
| Genotype A/A versus G/G | 1.3 | 0.23-7.01 | 0.54 |  | 0.5 | 0.11-2.43 | 0.27 |  | 0.8 | 0.19-3.40 | 0.79 |
| Genotype A/T versus G/G | 0.5 | 0.19-1.20 | 0.080 |  | 1.9 | 0.95-3.78 | 0.020 |  | 1.1 | 0.49-2.57 | 0.52 |
| Genotype G/A versus G/G | 1.0 | 0.37-2.65 | 0.63 |  | 1.1 | 0.53-2.24 | 0.88 |  | 0.9 | 0.39-1.97 | 0.87 |
| Genotype G/T versus G/G | 0.7 | 0.30-1.42 | 0.32 |  | 1.2 | 0.67-2.22 | 0.47 |  | 1.0 | 0.48-1.93 | 0.87 |
| Genotype T/T versus G/G | 0.9 | 0.37-2.26 | 0.79 |  | 1.0 | 0.50-2.03 | 0.88 |  | 0.8 | 0.38-1.83 | 0.71 |

Odds ratios for each genotype were adjusted for baseline eGFR, age, CD4 count, body weight, nephrotoxic drug use, hypertension, dyslipidemia, and use of PI/r. OR: odds ratio, CI: confidence interval, eGFR: estimated glomerular filtration rate, PI/r: ritonavir-boosted protease inhibitor.
