# Supplementary material for: Drug Transporter Genetic Variants Are Not Associated with TDF-Related Renal Dysfunction in Patients with HIV-1 Infection: A Pharmacogenetic Study
Source: PLoS One. 2015 Nov 4;10(11):e0141931. doi: 10.1371/journal.pone.0141931 (PMC4633171; doi:10.1371/journal.pone.0141931)
Supplement: S3 Table — (DOCX) [file pone.0141931.s003.docx]

S3 Table 3. Effects of SNPs on three renal endpoints using dominant, recessive, and additive genetic models for multivariate logistic analysis.

|  | >10 ml/min/1.73 m^2^ decrement in eGFR | | |  | >25% decrement in eGFR | | |  | eGFR <60 ml/min/1.73 m^2^ | | |
| --- | --- | --- | --- | --- | --- | --- | --- | --- | --- | --- | --- |
|  | OR | 95%CI | P value |  | OR | 95%CI | P value |  | OR | 95%CI | P value |
| -24 of *ABCC2* |  |  |  |  |  |  |  |  |  |  |  |
| Dominant model (genotype C/C + C/T versus T/T) | 0.4 | 0.06-3.63 | 0.45 |  | 1.1 | 0.40-3.00 | 0.86 |  | 0.6 | 0.19-1.97 | 0.41 |
| Recessive model (genotype C/C versus C/T + T/T) | 1.1 | 0.66-1.95 | 0.65 |  | 1.2 | 0.77-1.76 | 0.48 |  | 1.1 | 0.65-1.70 | 0.84 |
| Additive model | 1.0 | 0.64-1.69 | 0.87 |  | 1.1 | 0.79-1.61 | 0.51 |  | 1.0 | 0.64-1.49 | 0.92 |
| 1249 of *ABCC2* |  |  |  |  |  |  |  |  |  |  |  |
| Dominant model (genotype A/A + A/G versus G/G) | 0.7 | 0.40-1.38 | 0.35 |  | 1.0 | 0.59-1.55 | 0.86 |  | 1.0 | 0.56-1.73 | 0.94 |
| Recessive model (genotype A/A versus A/G + G/G) | 0.3 | 0.05-1.84 | 0.19 |  | 1.2 | 0.25-5.62 | 0.84 |  | 2.4 | 0.39-14.4 | 0.35 |
| Additive model | 0.7 | 0.41-1.24 | 0.23 |  | 1.0 | 0.63-1.51 | 0.91 |  | 1.0 | 0.62-1.75 | 0.87 |
| 2677 of *ABCB1* |  |  |  |  |  |  |  |  |  |  |  |
| Dominant model (genotype A/**^¶^**X versus **^*^**Y/Y) | 0.9 | 0.53-1.63 | 0.79 |  | 1.2 | 0.78-1.84 | 0.40 |  | 1.0 | 0.63-1.69 | 0.90 |
| Recessive model (genotype A/A versus others) | 1.7 | 0.34-8.30 | 0.53 |  | 0.4 | 0.10-1.90 | 0.27 |  | 0.8 | 0.21-3.27 | 0.79 |
| Additive model | 1.0 | 0.62-1.61 | 1.00 |  | 1.1 | 0.74-1.53 | 0.74 |  | 1.0 | 0.65-1.55 | 0.98 |

^¶^X denotes either A or T or G; ^*^Y denotes either T or G. Odds ratios for each genotype were adjusted for baseline eGFR, age, CD4 count, body weight, nephrotoxic drug use, hypertension, dyslipidemia, and use of PI/r. OR: odds ratio, CI: confidence interval, eGFR: estimated glomerular filtration rate, PI/r: ritonavir-boosted protease inhibitor.
